# Antioxidant Potential of the Bio-Based Fucose-Rich Polysaccharide FucoPol Supports Its Use in Oxidative Stress-Inducing Systems

**DOI:** 10.3390/polym13183020

**Published:** 2021-09-07

**Authors:** Bruno M. Guerreiro, Jorge Carvalho Silva, João Carlos Lima, Maria A. M. Reis, Filomena Freitas

**Affiliations:** 1 Associate Laboratory i4HB—Institute for Health and Bioeconomy, School of Science and Technology, NOVA University Lisbon, 2819-516 Caparica, Portugal; bm.guerreiro@campus.fct.unl.pt (B.M.G.); amr@fct.unl.pt (M.A.M.R.); 2 UCIBIO—Applied Molecular Biosciences Unit, Department of Chemistry, School of Science and Technology, NOVA University Lisbon, 2819-516 Caparica, Portugal; 3 CENIMAT/I3N, Department of Physics, NOVA School of Science and Technology, Universidade Nova de Lisboa, 2819-516 Caparica, Portugal; jcs@fct.unl.pt; 4 LAQV-REQUIMTE, Department of Chemistry, NOVA School of Science and Technology, Universidade Nova de Lisboa, 2819-516 Caparica, Portugal; lima@fct.unl.pt

**Keywords:** FucoPol, polysaccharide, antioxidant, stress, FRAP, Hill, hydrogen peroxide, food, radiation, cryopreservation

## Abstract

Reactive oxygen species (ROS) are dangerous sources of macromolecular damage. While most derive from mitochondrial oxidative phosphorylation, their production can be triggered by exogenous stresses, surpassing the extinction capacity of intrinsic antioxidant defense systems of cells. Here, we report the antioxidant activity of FucoPol, a fucose-rich polyanionic polysaccharide produced by *Enterobacter* A47, containing ca. 17 wt% of negatively charged residues in its structure. Ferric reducing antioxidant power (FRAP) assays coupled to Hill binding kinetics fitting have shown FucoPol can neutralize ferricyanide and Fe^3+^-TPTZ species at an EC_50_ of 896 and 602 µg/mL, respectively, with positive binding cooperativity (2.52 ≤ H ≤ 4.85). This reducing power is greater than most polysaccharides reported. Moreover, an optimal 0.25% *w*/*v* FucoPol concentration shown previously to be cryo- and photoprotective was also demonstrated to protect Vero cells against H_2_O_2_-induced acute exposure not only by attenuating metabolic viability decay, but also by accentuating post-stress proliferation capacity, whilst preserving cell morphology. These results on antioxidant activity provide evidence for the biopolymer’s ability to prevent positive feedback cascades of the radical-producing Fenton reaction. Ultimately, FucoPol provides a biotechnological alternative for implementation in cryopreservation, food supplementation, and photoprotective sunscreen formula design, as all fields benefit from an antioxidant functionality.

## 1. Introduction

Reactive oxygen species (ROS) such as the superoxide anion (O_2_^−^), hydrogen peroxide (H_2_O_2_) or hydroxyl radicals (HO^•^) are dangerous sources of macromolecular damage. Any cellular structure is subject to reactive degradation chemistry—lipids, DNA, and proteins alike [1]—and can become a source of further reactivity because ROS damage acts by a radicalization cascade [2] until cessation of radical species or antioxidant action.

The most frequent source of cellular ROS is of endogenous nature, as they are an inevitable consequence of mitochondrial oxidative phosphorylation [3], thus central to metabolism. However, exogenous stimuli can induce ROS overproduction such as chilling [4], drought [5], salinity [6], metal toxicity [7], pathogen invasion [8], and excessive ultraviolet (UV) exposure that leads to photolytic reactions [9].

Direct or indirect ROS-mediated macromolecular damage has been shown to be involved in several health conditions. Aging [10] is an inevitable consequence of baseline occurrences of ROS damage over the length of human life. Fast-food diets or consumption of products lacking antioxidant supplementation [11] often contribute to the development of atherosclerosis [12], diabetes [12], and neurodegeneration [13]. Negligent UV exposure often plays a role on the onset of carcinogenesis [14] (particularly, melanoma [15]) and premature skin photoaging [16].

FucoPol is a high-molecular-weight (1.7–5.8 × 10^6^ Da) fucose-containing polysaccharide secreted by the Gram-negative bacterium *Enterobacter* A47 (DSM 23139) [17,18], whose cryoprotective [19,20] and photoprotective [21] properties were recently demonstrated. FucoPol has a fucose, galactose, glucose, and glucuronic acid hexamer motif (2.0:1.9:0.9:0.5 M ratio), a main chain composed of a →4)-α-l-Fuc*p*-(1→4)-α-l-Fuc*p*-(1→3)-β-d-Glc*p*(1→ trimer repeating unit, and a trimer branch α-d-4,6-pyruvyl-Gal*p*-(1→4)-β-d-GlcA*p*-(1→3)-α-d-Gal*p*(1→ in the C-3 of the first fucose [19] (Figure S1). FucoPol also contains 13–14 wt% pyruvyl, 3–5 wt% acetyl, and 2–3 wt% succinyl in its acyl composition [22]. The presence of glucuronic acid as well as the acyl substituents pyruvyl and succinyl all confer a polyanionic character to the polymer [23]. Its aqueous solutions have a shear-thinning behavior reflective of a linearly-disposed helical conformation, with viscoelastic properties comparable to those of guar gum and fucogel [23]. Previous work has shown the multidisciplinary outreach of FucoPol applications. First, it acts as a crystallization inhibitor, successfully cryopreserving several animal cell lines [19,20]. Second, it has a rheological shear-thinning behavior [22] and emulsifying properties [18,23], which are highly desirable in the food industry. Recently, it has been shown to provide sunscreen-like photoprotective action to epithelial cells acutely exposed to UV radiation [21], a mechanism by which ROS cascades are also triggered. Such properties have been considered to benefit further from an additional antioxidant potential, bestowing enhanced performance to each field of application.

Cryoprotective formulas benefit from having antioxidant defenses to protect the cells against the chemical ROS outburst they undergo upon thawing: at cryogenic temperatures, diffusion is hindered and mitochondrial permeability is lost due to the absence of the regulatory proton gradient of ionic exchange [24], leaving the cell vulnerable to cytosolic radical attacks. In food products, supplementation with antioxidant substances is commonly desirable as poor dietary habits are often of chronic nature, which leads not only to diverse chronic cardiovascular conditions, but dysregulation of liver function and systemic inflammation mechanisms [25]. Finally, the exposure to ultraviolet radiation fundamentally results in photolytic transformations that lead to ROS production [26], and some polysaccharides have already shown radiation-protective effects that were associated with an antioxidant activity [27,28].

In this work, we studied the Fe^3+^ and H_2_O_2_ reductive power of FucoPol as a measure of its antioxidant activity. If proven, its use would alleviate the oxidative stress in biological systems and provide a solution to settings such as photoinduced damage during sun exposure, the oxidative outburst during thawing of cryopreservation material, and several other instances where ROS-induced macromolecular damage is prone to occur. Iron-species reduction was studied by FRAP and TPTZ assays, results were contrasted with water-soluble vitamins C and E, and radical-polysaccharide binding kinetics were modeled with the Hill equation for calculating the effective concentration (EC_50_) and cooperativity coefficient (H) in dose–response curves. Then, in vitro performance was assessed by inducing acute ROS production in Vero epithelial cells by exposure to concentrated H_2_O_2_ to mimic the ROS-mediated apoptotic cascade that occurs 6 h post-thawing during a cell cryopreservation procedure [29].

We have previously demonstrated that FucoPol can protect cells from freezing damage [19,20], but the absence of cell apoptosis after 6 h post-thaw was suggestive of FucoPol also enacting ROS scavenging. Some photolytic mechanisms also relate to oxidative damage when radical species are formed, and FucoPol has shown good photoprotective properties [21]. Thus, we hypothesized that both these effects could relate to antioxidant propensity.

## 2. Materials and Methods

### 2.1. FucoPol

FucoPol was produced by cultivation of *Enterobacter* A47 (DSM 23139) in a 2 L bioreactor (BioStat B-plus, Sartorius, Göttingen, Germany) under a fed-batch mode, using glycerol (Sigma-Aldrich, Germany) (40 g/L) as the carbon source, according to the procedure previously described [30]. FucoPol was extracted from the cultivation broth by dia/ultrafiltration, as previously described [31] and characterized in terms of sugar monomers and acyl group compositions, and molecular mass distribution, as previously described [30]. The sample had number (M_n_) and weight average molecular (M_w_) weights of 1.9 × 10^6^ Da and 3.3 × 10^6^ (±0.3 × 10^6^) Da, respectively, with a polydispersity of 1.70. Before in vitro usage, FucoPol was sterilized by dry autoclaving at 121 °C, 30 min, in a sealed Schott flask.

### 2.2. Evaluation of Antioxidant Activity

#### 2.2.1. Potassium Ferricyanide Assay

The ferric reducing antioxidant power (FRAP) of FucoPol was determined using an adaptation of the original method [32]. To a test tube, 250 µL of 200 mM cold PBS pH 6.6, 250 µL of 1% *w*/*v* K_3_Fe(CN)_6_ (Sigma-Aldrich, Germany), and 250 µL of the test sample were added. The mixture was briefly vortexed until homogenized and then left to incubate at 50 °C for 20 min. Then, 250 µL of 10% *w*/*v* trichloroacetic acid (TCA) (Sigma-Aldrich, Germany) was added. The mixture was centrifuged at 2375× *g* for 10 min and a biphasic system formed. To the collected upper layer, 50 µL of 0.1% (*w*/*v*) FeCl_3_ (Sigma-Aldrich, Germany) (freshly prepared, dark storage) was added. After 10 min of reaction time, absorbance at 700 nm was measured. FucoPol solutions were prepared in distilled water at concentrations of 0.1, 0.08, 0.06, 0.04, 0.02, 0.01, and 0.005% (*w*/*v*). Distilled water was used as the blank and ascorbic acid (vitamin C), of equal concentration range, as the positive control.

#### 2.2.2. 2,4,6-Tris(2-pyridyl)-s-triazine (TPTZ) Assay

For validation, a second FRAP determination of FucoPol was performed with an adaptation of the original method [33]. A working FRAP solution (WFRAP) was prepared by mixing 25 mL of 0.3 M cold acetate buffer, 2.5 mL of 0.01 M cold 2,4,6-tris(2-pyridyl)-s-triazine (TPTZ, dark storage) dissolved in 0.04 M HCl, and 2.5 mL of 0.02 M FeCl_3_·6H_2_O (freshly prepared, dark storage). After homogenization of the initial mix, a 270 µL aliquot was collected, to which 27 µL of distilled water and 9 µL of test sample were added. The mixture was quickly vortexed, incubated at 37 °C for 30 min, and absorbance at 595 nm was measured. FucoPol solutions were prepared at concentrations of 0.1, 0.08, 0.06, 0.04, 0.02, 0.01, and 0.005% (*w*/*v*). Distilled water was used as the blank and Trolox (water-soluble analog of vitamin E) at concentrations 300, 250, 200, 150, 100, and 50 µM was used as the positive control. All reagents purchased from Sigma-Aldrich (Germany).

#### 2.2.3. Determination of Binding Kinetics

The determination of the effective concentration required to produce 50% of the total antioxidant response (EC_50_) and the corresponding Hill coefficient of binding interaction (H) were estimated according to the following equation [34]:

(1) $$y=A_{min}+\frac{A_{max}-A_{min}}{1+{\left(\frac{x}{{\mathrm{EC}}_{50}}\right)}^{-H}}$$

where *A_min_* and *A_max_* are the lowest and highest absorbance value, respectively, collected for the given concentration range.

#### 2.2.4. In Vitro H_2_O_2_-Mediated Oxidative Stress Induction

Vero cells were seeded in 96-well microplates at a density of 20,000 cells/well and underwent a reactive-oxygen species (ROS) induction assay at 37 °C, as previously described [35]. Vero cells were pre-incubated in DMEM alone or with 0.25% (*w*/*v*) FucoPol, for 1 h, and subsequently exposed to 300 µM of H_2_O_2_ for 3 and 6 h. Two negative control conditions (one for each medium) of non-exposure to H_2_O_2_ were also performed under the same conditions. For assessment of the cells’ metabolic viability, 0.1 volumes of 5 mg/mL of 3-(4,5-dimethylthiazol-2-yl)-2,5-diphenyltetrazolium bromide (MTT) dissolved in pure DMSO (room-temperature, dark storage) were added after sterilizing the solution with 0.2 µm filters. After incubating for 3 h at 37 °C, the liquid content of each well was discarded and 100 µL of pure DMSO was added. Absorbance at 570 and 690 nm was measured and subtraction of both values, respectively, yielded the proportional metabolic activity. All reagents purchased from Sigma-Aldrich (Germany).

### 2.3. Optical Microscopy Image Collection

A NIKON Eclipse Ti-S optical microscope equipped with a NIKON D610 digital camera (Nikon Corporation, Tokyo, Japan) was used. Pictures were collected with the Camera Control Pro software (Nikon Corporation, Japan). Images taken show the typical morphology of cells and are not intended for cell quantification.

### 2.4. Statistical Analysis

All experiments are triplicates and results are expressed as mean ± experimental standard deviation (SD). Statistical significance was analyzed with two-tailed student’s *t*-test, assuming a 95% confidence interval. Statistical designations on top of each bar show the significance level and correspond to *p*-value thresholds of 0.1234 (ns), 0.0332 (*), 0.0021 (**), 0.0002 (***), and <0.0001 (****). Normality of data was assessed with the Anderson–Darling, D’Agostino and Pearson, Shapiro–Wilk, and Kolmogorov–Smirnov normal distribution tests. To assess sample size, additional investigation of the effect size was performed with quantile–quantile (Q–Q) plots.

## 3. Results

### 3.1. FRAP Assays

**Figure 1:**
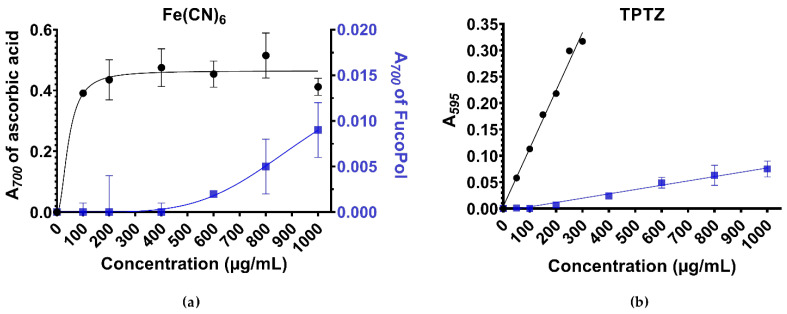
Antioxidant properties of the polysaccharide FucoPol (blue squares), presented as mean absorbance of the final reaction product as a function of concentration. Concentrations represent effective concentration of a molecule in aqueous medium, and do not factor in reactional mixture dilutions. (**a**) FRAP assay using ferricyanide as oxidant species and ascorbic acid (vitamin C) as positive control (black circles). Best-fit curves are shown to demonstrate tendency of change. Best fit for FucoPol was a second-order polynomial, *y* = 10^−8^*x*^2^ − 5 × 10^−6^*x* (R^2^ = 0.993). (**b**) FRAP assay using TPTZ as the oxidant species and Trolox (water-soluble vitamin E analog) as the positive control (black). Best fit for FucoPol was a first-order polynomial, *y* = 8.19 × 10^−5^*x* − 0.004872 (R^2^ = 0.9841).

In the ferricyanide assay (Figure 1), FucoPol presented a dose-dependent increase in its ability to perform the Fe(III) → Fe(II) reduction. FucoPol started to be sensitive to the presence of Fe^3+^ at 400 µg/mL. In contrast, ascorbic acid rapidly achieved a plateau at a reducing power an order of magnitude greater. Linear and polynomial fits were performed to the 400–1000 µg/mL range of FucoPol, with the second-order polynomial equation best describing its behavior with a correlation factor (R^2^) of 0.9934 compared with 0.9646 for the linear fit.

The ferricyanide assay is known for its low sensitivity at the concentration range used [36]. Thus, the TPTZ assay was performed as validation of the previous results (Figure 1). A dose-dependent increase was also observed, although in a linear fashion, thus confirming that the antioxidant activity of FucoPol was significant, but Trolox showed greater efficiency in reducing Fe^3+^-TPTZ. Once again, a measurable reducing power could be observed from 400 µg/mL FucoPol onwards, although it seems the polysaccharide is already sensitive to the presence of Fe^3+^ at 200 µg/mL (or, ppm). This minimum concentration threshold range has similarly been documented for other polysaccharides and appears to be an intrinsic measure of responsiveness for high-molecular weight molecules [33,37,38].

In the ferricyanide assay, ascorbic acid and FucoPol showed a reductive power of 0.412 and 0.009 absorbance units, respectively, for the highest concentration studied (1000 μg/mL). In the TPTZ assay, Trolox and FucoPol showed a reductive power of 0.317 and 0.075 absorbance units, respectively, for their corresponding highest concentrations studied (300 and 1000 μg/mL). As a comparative measure, FucoPol at 300 μg/mL was calculated, by means of linear regression, to have a reductive power of 0.020 absorbance units.

Without considering the effects of hindered microradical diffusion through the viscous medium that FucoPol originates at increasing concentrations, the low absorbance values observed for FucoPol in both assays can also be interpreted as a molar imbalance. For instance, equipercentual concentrations of substances of exceptionally different molecular weight imply that ascorbic acid molarity is, at least, three orders of magnitude higher than the molar concentration of FucoPol for a given concentration value, so the amount of reactive ascorbic acid was much higher than the polysaccharide molecules. The same logic applies for Trolox. Because chain entanglement or steric hindrance effects are unlikely at this concentration range [39], a plausible explanation for reduced absorbance values might also be the proportion between the reactive polar groups and the structural neutral sugars in the polymer chain

Previous work on this polysaccharide showed that outstanding cryoprotective and photoprotective potential could be observed with an optimal 0.25% FucoPol concentration [19,21]. Thus, we extrapolated the antioxidant potential of FucoPol at this concentration by using the FRAP-based modeling previously obtained. In the ferricyanide assay (Figure 1a), the second-order polynomial best fit *y* = 10^−8^*x*^2^ − 5 × 10^−6^*x* (R^2^ = 0.993) was used to estimate the antioxidant power. By extrapolation, a 0.25% FucoPol solution was 5.6 times more potent than the highest concentration studied, 0.1% FucoPol. If a horizontal intersecting line is drawn upon the graph of Figure 1a, that value is equivalent to approximately the antioxidant power of 11 µg/mL (0.06 mM) ascorbic acid. Similarly, in the TPTZ assay (Figure 1b), the first-order polynomial *y* = 8.19 × 10^−5^*x* − 0.004872 (R^2^ = 0.9841) was used to estimate that a 0.25% FucoPol solution has an antioxidant power 2.7-fold higher than 0.1% FucoPol, equivalent to 176 µg/mL (0.7 mM) of Trolox.

### 3.2. Determination of Hill Binding Kinetics

**Figure 2:**
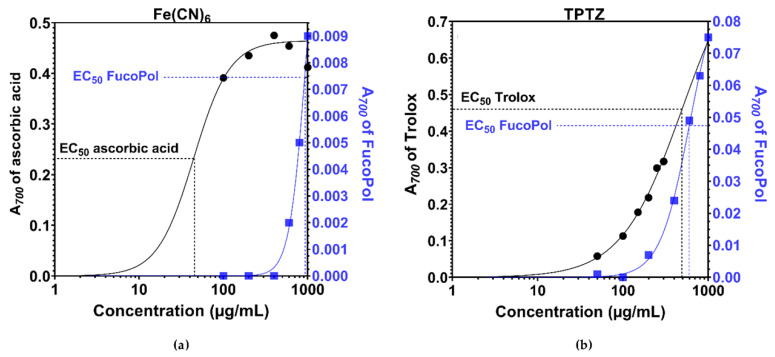
Model fitting of Hill binding kinetics. EC_50_ and Hill slope was determined using Equation (1) for both FRAP assays using (**a**) ferricyanide and (**b**) TPTZ mean absorbance data. Data for FucoPol is represented in blue (squares).

The Hill Equation (1) was used to determine the effective concentration at which 50% of Fe^3+^-species are reduced (EC_50_) and its corresponding Hill coefficient (H) as a measure of binding interaction and cooperativity in dose–response curves [40] (Figure 2). Application of the Hill equation to antioxidant dose–response curves provides a sufficiently accurate fit to describe radical–polysaccharide binding interactions if we consider at first, by simplicity, that binding is independent of external factors (e.g., no multiple ligand binding or multimeric assemblies occur). It has been shown that non-neutral cooperativity can occur in single-ligand binding receptors, because cooperative interactions are an inherent consequence of the dynamic structural rearrangements of macromolecules that accompany ligand binding [41].

The {EC_50_, H} variable pair was determined for both assays performed. In the ferricyanide assay, ascorbic acid had {44.4 µg/mL, 2.03} and FucoPol {896.0 µg/mL, 4.85}. In the TPTZ assay, Trolox had {490.4 µg/mL, 1.21} and FucoPol {602.1 µg/mL, 2.52}.

The collected EC_50_ values show, on one hand, that ascorbic acid and Trolox have 20.2 and 1.2-fold higher affinity than FucoPol, respectively, in scavenging 50% of the Fe^3+^-species. On the other hand, FucoPol showed a Hill coefficient 2.4 and 2.1-fold higher than ascorbic acid and Trolox, as corroborated by steeper dose–response slopes. This suggests that whilst there is a delayed responsiveness of FucoPol to radical species in the microenvironment, greater positive cooperativity between receptor binding sites and the oxidant molecule is observed when reductive activity spurs. The literature suggests that ascorbic acid reacts with Fe^3+^-species in a 1:2 stoichiometry [42], shows a sigmoidal dose–response curve, and a Hill coefficient of two characteristics of positive cooperativity [39,43]. This is corroborated by the binding coefficient (H = 2.03) obtained here.

FucoPol showed strong positive cooperativity within the interval 2.52 ≤ H ≤ 4.85. According to the Hill equation, H can be correlated with receptor–ligand stoichiometry. For a 1:n ratio, H ranges from [0, n]. For positive cooperativity (H > 1, in which the binding of a first ligand facilitates the binding of a second in adjacent free receptors), H varies within [1, n], with n being the upper limit of maximal cooperativity because the product n × H cannot be larger than the number of available binding sites. Given that almost one-fourth of FucoPol polymer weight is composed of negatively charged acyl groups randomly distributed throughout the polymeric chain, the H coefficient was expected to be higher, but allosteric effects decreased exponentially over distance [37,41], most likely downsizing this value to what was obtained.

Once again, from extrapolation of the binding kinetics for 0.25% *w*/*v* FucoPol, we deduce that this concentration accounts for 8.2 µg/mL (0.044 mM) of ascorbic acid and 81.3 µg/mL (0.32 mM) of Trolox, in terms of equivalent reductive potential.

Ultimately, in a scenario of acute oxidative damage, the ability of FucoPol to quench radicals will vary exponentially over time as its structural compliance to minimize binding energy upon sequential binding will neutralize oxidative species faster than an oxidant that only acts by independent binding, with a linear tendency characterized by H = 1. Additionally, both similarities of (i) ascorbic acid binding kinetics to previous descriptions in the literature [39,43] and (ii) FucoPol Hill-based to FRAP-based extrapolations suggest that antioxidant data analysis using the Hill equation is a plausible approach in radical–antioxidant dose–response curves.

### 3.3. FucoPol In Vitro Scavenging Potential of H_2_O_2_-Derived Species

The extrapolations of reducing power to 0.25% FucoPol and its previously observed cryoprotective and photoprotective effects at this concentration [19,21] all supported the postulation of in vitro antioxidant performance. To substantiate the claim that reducing power can be translated to cell protection from oxidative damage, an oxidative damage induction procedure was performed in the presence of 0.25% FucoPol.

In cryopreservation, necrotic and apoptotic cascades have been shown to occur in the first 6 h of post-thaw metabolism after apparently successful preservation [38]. By exposing Vero cells to H_2_O_2_, acute ROS production can be induced and the oxidative outburst a cell undergoes after thawing is mimicked. This mechanism of cell damage is transversal and equally important to photobiology, as delayed loss of homeostasis due to UV exposure can also occur [44].

**Figure 3:**
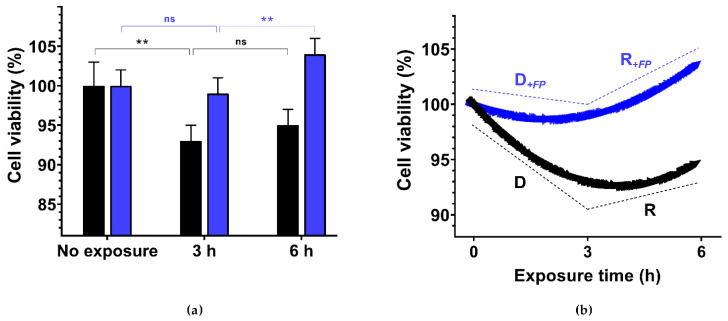
Vero epithelial cell metabolic viability (**a**) and its corresponding temporal evolution (**b**) post-ROS induction with an acute H_2_O_2_ concentration. Viability is shown as mean absorbance data, in the presence (blue) or absence (black) of FucoPol for non-exposed cells, and cells exposed to 300 µM H_2_O_2_ for 3 and 6 h. The corresponding evolution of viability over time, shown as decay–recovery response curves, were modeled with the following linear segments: D (*y* = −2.33*t* + 100), D*_+FP_* (*y* = −0.33*t* + 100), R (*y* = +0.67*t* + 91), and R*_+FP_* (*y* = +1.67*t* + 94). Two-tailed *t*-student tests between time points were employed to check for statistical significance. Data were normality distributed, according to Anderson–Darling, D’Agostino and Pearson, Shapiro–Wilk, and Kolmogorov–Smirnov normality tests. The ** symbol represents *p* < 0.0021 statistical significance for 95% CI.

Figure 3 shows the temporal evolution of Vero cell metabolic viability when exposed to 300 µM H_2_O_2_ in the control medium DMEM or in the presence of 0.25% FucoPol (for normality tests see Figure S2 and Table S1; for *t*-student tests see Table S2). In DMEM, cell viability dropped to 93 ± 2% after being exposed to H_2_O_2_ for 3 h, slightly recovering to 95 ± 2% after 6 h of exposure (Figure 3, black bars). In the presence of 0.25% FucoPol, there was no significant decrease in cell viability after 3 h of exposure to H_2_O_2_ (99 ± 2% compared to 93 ± 2% for the unprotected cells), while cell proliferation was higher 104 ± 2% after 6 h (Figure 3, blue bars). The overall trend of metabolic viability in both conditions is also outlined in the rightmost plot. The addition of 0.25% FucoPol not only resulted in a 7-fold attenuation of the decay of viability, but the recovery of proliferation was accentuated 2.5-fold in comparison to the control conditions.

**Figure 4:**
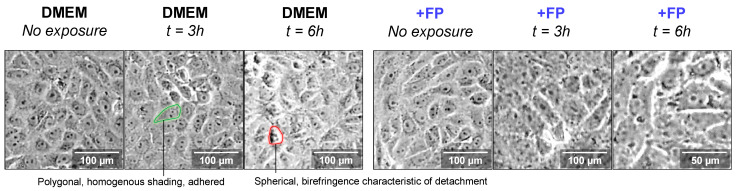
Temporal evolution of Vero cell morphology post-ROS induction with acute H_2_O_2_ concentration. Morphologies in the absence of an antioxidant (DMEM) and in the presence of 0.25% FucoPol (+FP) were collected with a 100× magnification. Notice the absence of birefringence characteristic of cell detachment as a consequence of death. Green indicates a healthy cell, red indicates a dying cell. This evolution is corroborated by the observation of cell morphology (Figure 4). In the sole presence of DMEM, non-exposed Vero cells presented a polygonal aspect, congruent with good cell adherence and function, but there is visible cell detachment with increased exposure time, characteristic of cell death (Figure 4, DMEM). In the presence of 0.25% FucoPol, the healthy attributes of Vero cell morphology were preserved over time, particularly the degree of attachment and cell–cell interactions.

This evolution is corroborated by the observation of cell morphology (Figure 4). In the sole presence of DMEM, non-exposed Vero cells presented a polygonal aspect, congruent with good cell adherence and function, but there is visible cell detachment with increased exposure time, characteristic of cell death (Figure 4, DMEM). In the presence of 0.25% FucoPol, the healthy attributes of Vero cell morphology are preserved over time, particularly the degree of attachment and cell-cell interactions. 

These results are in agreement with previous documentation on other fucose-rich polysaccharides. Chowdhury et al. [35] performed antioxidant and anti-apoptotic studies on the fucose-containing exopolysaccharide produced by *Bacillus megabacterium* RB05 and demonstrated that under identical acute oxidative stress, the polysaccharide provided a direct scavenging effect. Specifically, the EC_50_ for H_2_O_2_ scavenging was determined to be 1500 µg/mL. In our work, we used a concentration of 2500 µg/mL FucoPol, which, according to their dose–response curve, would account for ca. 70% of scavenged H_2_O_2_, assuming equivalent EC_50_.

## 4. Discussion

The Fenton reaction [1] is a dangerous Fe^2+^-catalyzed process that converts H_2_O_2_, a byproduct of mitochondrial oxidative respiration, into highly reactive HO^•^ radicals. The oxidized Fe^3+^ can then react again with H_2_O_2_ to form a second toxic molecule, the perhydroxyl radical HOO^•^, which is highly damaging to cellular structures by promoting the continuous progression of current oxidative cascades and the development of new ones. FucoPol is a polyanionic polysaccharide because it contains approximately 22 wt% of negatively charged succinyl, pyruvyl, and glucuronic acid groups in its structure [22]. Its negatively charged residues can then participate in redox reactions and scavenge oxidative radicals at physiological pH, thus maintaining cellular homeostatic balance. Thus, the antioxidant activity of FucoPol was studied with ferric reducing antioxidant power (FRAP) assays and an in vitro oxidative induction experiment, in an attempt to understand how FucoPol could interfere with the Fenton pathway.

A FRAP assay tests the ability of a substance to reduce ferric species into its ferrous counterparts, at the expense of test substance oxidation [45]. This converts directly into an antioxidant power that can be measured by binding capacity and affinity. Ferricyanide and ferric TPTZ were used as the oxidant species. The in vitro assay was based on acute ROS induction on living cells using H_2_O_2_. A practical example would be the mimicking of the ROS-mediated apoptotic cascade that occurs 6 h post-thawing during a cell cryopreservation procedure [29], and it reflects the ability of FucoPol to extinguish the generated ROS, and its influence on cell proliferation and morphology after the acute event.

FucoPol was previously shown to act as an antioxidant activity enhancer of gallic acid and oregano oil [33], but its individual antioxidant contribution remained unknown. In this study, we have shown its promising reducing power toward Fe^3+^ species, an especially high Hill coefficient characteristic of good affinity toward oxidant molecules, and the power to protect Vero cells against H_2_O_2_ oxidative induction, therefore showing the potential to prevent the nefarious effects of the Fenton cascade. Additionally, the EC_50_ data now suggests that FucoPol has a greater Fe(III)-reducing power than other exopolysaccharides reported in the literature (Table 1), pointing to its promising use as an antioxidant carrier and/or active ingredient.

However, its mechanism of action remains to be elucidated, although some hypotheses can be postulated. The polysaccharide can act by a (i) direct ROS scavenging effect, (ii) a strong chelating effect on metal ions, or (iii) a reductive potential balanced by self-oxidation [48]. The first two hypotheses were the focus of this study, and the data suggest that FucoPol directly interacts with ferrous ions and chelates them, as seen in FRAP assays, but also has the potential to scavenge ROS, whereas an increased viability of cells in the H_2_O_2_ exposure assay points to the efficiency of FucoPol in inhibiting the progression of damaging Fenton reactions. The third hypothesis is probable but unlikely, because it would imply a cumulative loss of antioxidant potential due to structural changes by self-oxidation, and we have recently shown [21] that FucoPol shows near-complete regeneration of its original structure after exposure to UV radiation that is strong enough to produce damaging radicals and induce photodegradation. If there is such an effect, the contribution of self-oxidation to its antioxidant power is minimal.

Regardless, the effectiveness of FucoPol in reducing the cellular stress response by eliminating the threat beforehand is evident, thus providing a healthier microenvironment for cell proliferation. This has implications in very diverse fields that have oxidative stress as a common factor, ranging from age-related diseases, diabetes, cardiovascular diseases, and regenerative medicine to food supplementation, biopreservation, and dermatologic formulations such as sunscreens and cosmetics. In all these applications, FucoPol can be a bio-based supplement to suppress oxidative damage and guarantee cellular health.

## 5. Conclusions

The fucose-rich polysaccharide FucoPol was studied under the assumption that its high content of negatively charged groups could provide significant antioxidant potential. FucoPol showed a dose-dependent capacity to reduce two distinct Fe^3+^ species and successfully neutralized an H_2_O_2_-induced acute exposure event in vitro, showing promising neutralization efficacy against post-respiratory Fenton reactions. Dose–response Hill fitting unveiled that FucoPol had a lower EC_50_ than most antioxidant bio-based polysaccharides reported and showed a positive cooperativity behavior, suggesting an exponentially reduced time of action required to neutralize reactive species in the medium. An optimal 0.25% FucoPol concentration used in cryopreservation and photoprotective settings also showed sterling performance in vitro. Not only did it attenuate the viability decay of Vero cells acutely exposed to H_2_O_2_, but also accentuated their post-stress proliferation capacity, whilst preserving cell morphology. Ultimately, FucoPol provides a biotechnological alternative for implementation in cryopreservation, food supplementation, and photoprotective cosmetics.

## Figures and Tables

**Table 1:** Literature collection of antioxidant activity observed in polysaccharides from different sources. Different experiments represent different sources of data that are not fully comparable. Fe^3+^ reduction assays refer to contrasts with ascorbic acid, unless referred otherwise.

Polymer (M_w_)	Strain	Assay	EC_50_	Ref.
Glucose and rhamnose-rich EPS (10^5^ Da)	*Weisella cibaria* GA44	DPPH Fe^3+^ reduction O_2_^•−^ scavenging	4000 µg/mL 2000 µg/mL 1200 µg/mL	[46]
Starch-like EPS (10^4^ Da)	*Peanibacillus mucilaginosus* TKU032	DPPH Fe^3+^ reduction	157 µg/mL n/d (44% = ca. 500 µg/mL)	[47]
Fructose-rich EPS (10^6^ Da)	*Paenibacillus polymyxa* EJS-3	Fe^3+^ reduction O_2_^•−^ scavenging OH^•^ scavenging	n/d (44% = ca. 1000 µg/mL) 400 µg/mL 220 µg/mL	[32]
FucoPol (1–6 × 10^6^ Da)	*Enterobacter* A47	Fe^3+^ reduction Fe^3+^ reduction	896 µg/mL (Ascorbic acid) 602 µg/mL (Trolox)	This work

## Data Availability

The data presented in this study are available on request from the first author or corresponding author.

## Supplementary Materials

The following are available online at https://www.mdpi.com/article/10.3390/polym13183020/s1, Table S1: Results from Q–Q normality tests on the control and test conditions from data in Figure 3a, Table S2: Student’s *t*-test results from time point comparisons of data in Figure 3a, Figure S1: Q–Q normality plot of control and test conditions from data in Figure 3a, Figure S2: Tentative structure of FucoPol based on preliminary experimental data. The structure presented is a deacetylated, desuccinylated form of the biopolymer.
