# Supplementary material for: Antioxidant Potential of the Bio-Based Fucose-Rich Polysaccharide FucoPol Supports Its Use in Oxidative Stress-Inducing Systems
Source: Polymers (Basel). 2021 Sep 7;13(18):3020. doi: 10.3390/polym13183020 (PMC8470694; doi:10.3390/polym13183020)
Supplement: Supplementary file 1 [file polymers-13-03020-s001.zip › polymers-1341614-supplementary.pdf]

Supplementary Materials to:

Antioxidant potential of the bio-based fucose-rich polysaccharide FucoPol supports its use in oxidative stress-inducing systems

Bruno M. Guerreiro[1,2,3,4], Jorge C. Silva[3], João C. Lima[4], Maria A. M. Reis[1,2], Filomena Freitas[1,2*]

[1] Associate Laboratory i4HB - Institute for Health and Bioeconomy, School of Science and Technology, NOVA University Lisbon, Caparica, Portugal; bm.guerreiro@campus.fct.unl.pt, amr@fct.unl.pt; a4406@fct.unl.pt

[2] UCIBIO – Applied Molecular Biosciences Unit, Department of Chemistry, School of Science and Tech-nology, NOVA University Lisbon, Caparica, Portugal; bm.guerreiro@campus.fct.unl.pt, amr@fct.unl.pt; a4406@fct.unl.pt

[3] CENIMAT/I3N, Department of Physics, NOVA School of Science and Technology, Universidade Nova de Lisboa, 2819-516 Caparica, Portugal; jcs@fct.unl.pt

[4] LAQV-REQUIMTE, Department of Chemistry, NOVA School of Science and Technology, Universidade Nova de Lisboa, 2819-516 Caparica, Portugal; lima@fct.unl.pt

* Correspondence: a4406@fct.unl.pt; Tel.: (+351212948300, ext: 10947)

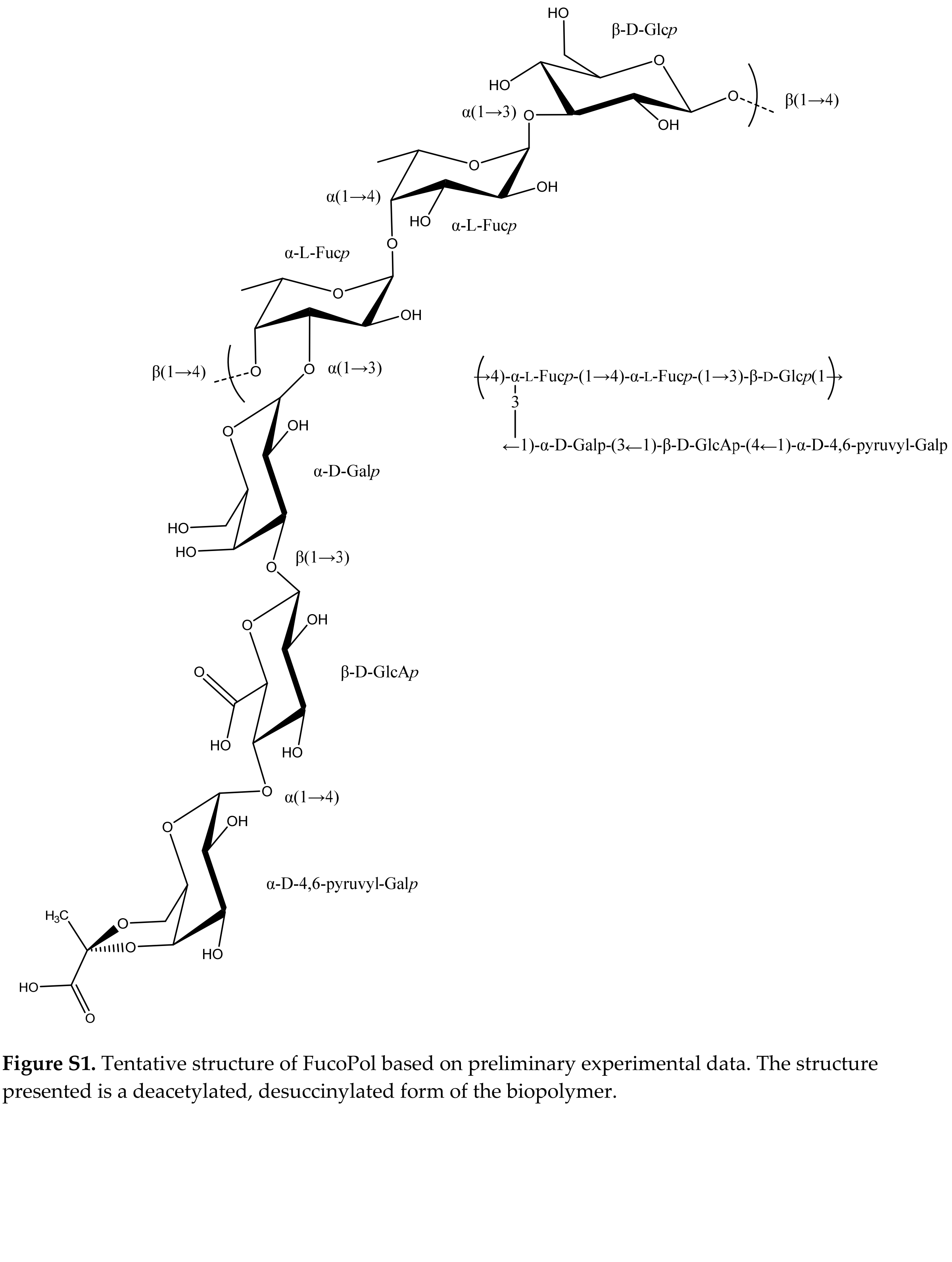

Figure S1. Tentative structure of FucoPol based on preliminary experimental data. The structure presented is a deacetylated, desuccinylated form of the biopolymer.

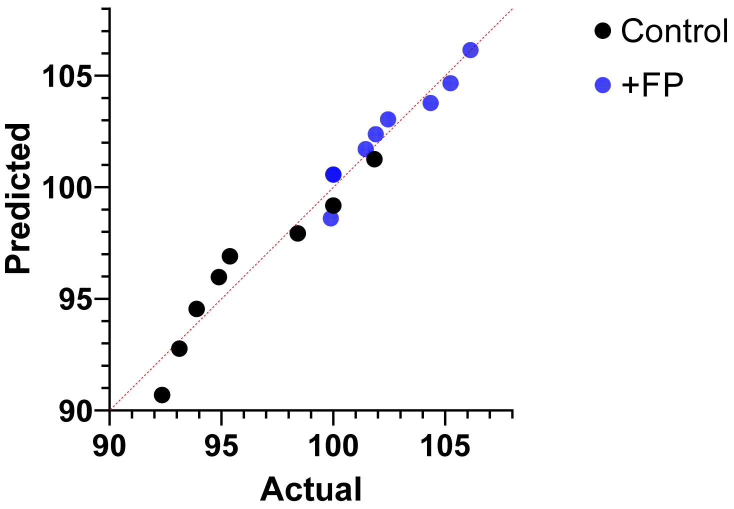

Figure S2. Q-Q normality plot of control and test conditions from data in Figure 3a.

Table S1. Results from Q-Q normality tests on the control and test conditions from data in Figure 3a.

Parameters	*Control*	*+FP*
**Test for normal distribution**		
**Anderson-Darling test**		
A2*	0.4789	0.3914
P value	0.1738	0.2999
Passed normality test (alpha=0.05)?	Yes	Yes
P value summary	ns	ns
**D'Agostino & Pearson test**		
K2	1.560	1.516
P value	0.4583	0.4686
Passed normality test (alpha=0.05)?	Yes	Yes
P value summary	ns	ns
**Shapiro-Wilk test**		
W	0.8906	0.8952
P value	0.2022	0.2257
Passed normality test (alpha=0.05)?	Yes	Yes
P value summary	ns	ns
**Kolmogorov-Smirnov test**		
KS distance	0.2378	0.1761
P value	>0.1000	>0.1000
Passed normality test (alpha=0.05)?	Yes	Yes
P value summary	ns	ns
**Number of values**	9	9

Table S2. Student's *t*-test results from time point comparisons of data in Figure 3a.

Parameters	*Control*		*+FP*	
	0h *vs* 3h	3h *vs* 6h	0h *vs* 3h	3h *vs* 6h
**Unpaired t test**				
P value	0.0030	0.0625	0.5065	0.0050
P value summary	**	ns	ns	**
Significantly different (P < 0.05)?	Yes	No	No	Yes
One- or two-tailed P value?	Two-tailed	Two-tailed	Two-tailed	Two-tailed
t, df	t=6.411, df=4	t=2.562, df=4	t=0.7287, df=4	t=5.591, df=4
**How big is the difference?**				
Mean of column A	100.1	93.12	101.3	100.6
Mean of column B	93.12	94.72	100.6	105.2
Difference between means (B - A) ± SEM	-6.963 ± 1.086	1.600 ± 0.6244	-0.7033 ± 0.9651	4.643 ± 0.8305
95% confidence interval	-9.979 to -3.948	-0.1336 to 3.334	-3.383 to 1.976	2.338 to 6.949
R squared (eta squared)	0.9113	0.6214	0.1172	0.8866
**F test to compare variances**				
F, DFn, Dfd	4.970, 2, 2	1.028, 2, 2	1.188, 2, 2	1.612, 2, 2
P value	0.3350	0.9861	0.9140	0.7656
P value summary	ns	ns	ns	ns
Significantly different (P < 0.05)?	No	No	No	No
**Data analyzed**				
Sample size, column A	3	3	3	3
Sample size, column B	3	3	3	3
